# Pneumoperitoneum without Gastrointestinal Perforation in a Neonate with Esophageal Atresia

**Published:** 2014-10-20

**Authors:** Manoj Saha

**Affiliations:** Department of Pediatric Surgery, Gauhati Medical College, Guwahati, INDIA

**Dear Sir**

A 3-day-old male child was referred to us with the diagnosis of esophageal atresia (EA) and trachea-esophageal fistula (TEF). The baby was first child of the parents, born at term by caesarean section. Baby cried immediately after birth and there was no respiratory distress. As the child regurgitated the first feed, x-ray was taken with an infant feeding tube in situ and subsequently the child was sent to our facility for further care. His general condition was stable; there was mild respiratory distress but no cyanosis. Abdomen was distended and scrotum was tense cystic due to air. Repeat x-ray was taken and it showed gross pneumoperitoneum with air in the scrotum (Fig. 1). Right thoracotomy, fistula ligation and end to end esophageal anastomosis were done. Then laparotomy was done by a transverse upper abdominal incision. There was free air and small amount of peritoneal fluid but no perforation was found in entire gastrointestinal tract. Abdomen was closed with a peritoneal drain. Child could be extubated immediately after the operation. Post-operative period was uneventful. Bacterial culture of the peritoneal fluid was sterile. Drain was removed after 48 hours. Nasogastric feeding was started after 24 hours and oral feed started on 5th postoperative day. Child was discharged on 9th post operative day.

Pneumoperitoneum in newborn most commonly arises from perforated hollow viscus. Necrotizing enterocolitis (NEC) remains the single most common cause of pneumoperitoneum in neonates [1, 2]. Spontaneous pneumoperitoneum without hollow viscus perforation is rare in newborn and is usually associated with pulmonary air leak syndromes as pneumothorax and pneumomediastinum [3]. But pneumoperitoneum in absence of hollow viscus perforation and pulmonary air leak syndrome is very rare at any pediatric age. This phenomenon has been reported in a neonate of 36 weeks gestation by Shah et al [4] and also in neonate of 34 weeks gestation with hyaline membrane disease who developed pneumoperitoneum after recovery from respiratory distress [5]. In another retrospective analysis of 89 neonates with pneumoperitoneum, cause of pneumoperitoneum could not be ascertained in 7 cases [1]. Exclusion of bowel perforation is crucial in cases of neonatal pneumoperitoneum as missed intestinal perforation would certainly compromise them further. Our patient was a term baby, without respiratory distress and pulmonary air leak and was not ventilated. The child developed pneumoperitoneum on third day of life. Contrast x-ray was not possible and the cause of the pneumoperitoneum remained obscure.

**Figure:**
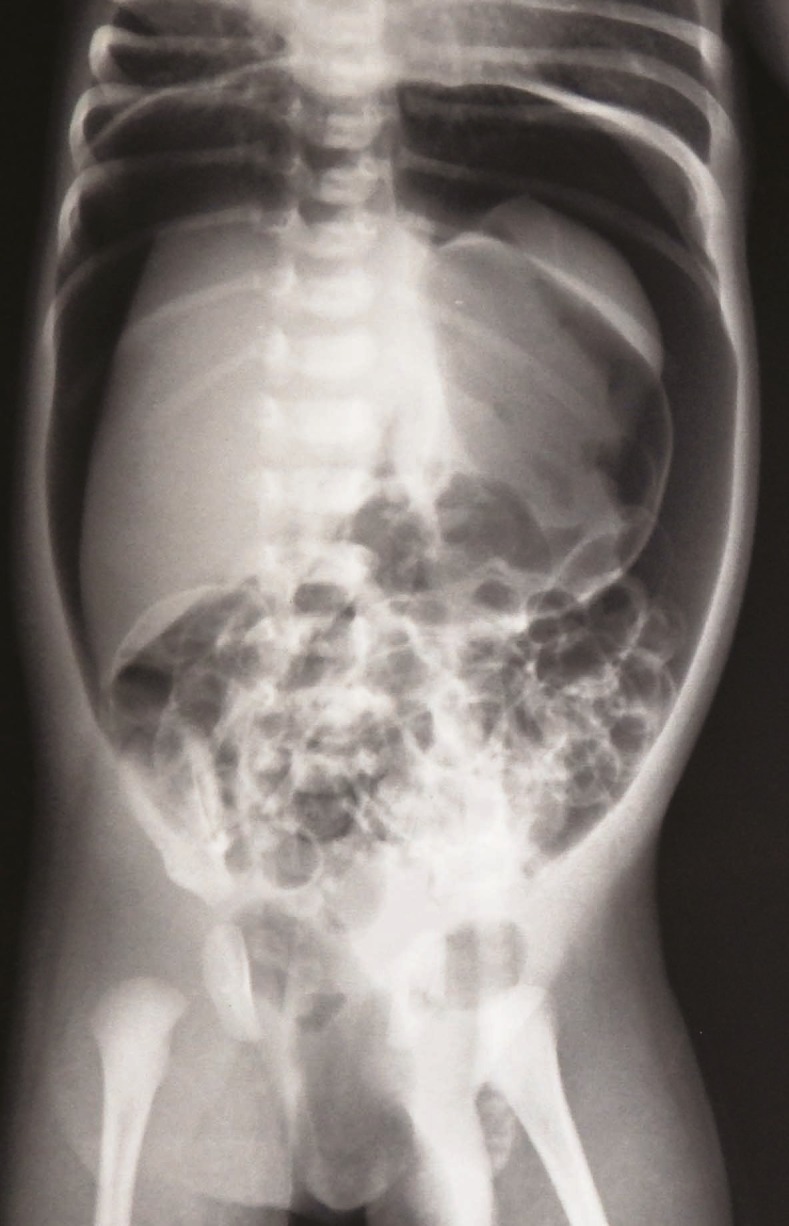
Figure 1: Gross pneumoperitoneum with air in the scrotum.

## Footnotes

**Source of Support:** Nil

**Conflict of Interest:** None

